# Early identification of patients requiring massive transfusion, embolization, or hemostatic surgery for traumatic hemorrhage: a systematic review protocol

**DOI:** 10.1186/s13643-017-0480-0

**Published:** 2017-04-13

**Authors:** Alexandre Tran, Maher Matar, Ewout W. Steyerberg, Jacinthe Lampron, Monica Taljaard, Christian Vaillancourt

**Affiliations:** 1 School of Epidemiology, Public Health and Preventive Medicine, University of Ottawa, Ottawa, Ontario Canada; 2 Division of General Surgery, The Ottawa Hospital, The Ottawa Hospital Civic Campus, Loeb Research Building, Main Floor, 725 Parkdale Avenue, Office WM150E, Ottawa, Ontario K1Y 4E9 Canada; 3 Department of Medical Statistics and Bioinformatics, Leiden University Medical Center, Leiden, The Netherlands; 4 Clinical Epidemiology Program, Ottawa Hospital Research Institute, Ottawa, Canada; 5 Department of Emergency Medicine, University of Ottawa, Ottawa, Canada

**Keywords:** Traumatic hemorrhage, Prediction model, Massive transfusion, Surgery, Embolization

## Abstract

**Background:**

Hemorrhage is a major cause of early mortality following a traumatic injury. The progression and consequences of significant blood loss occur quickly as death from hemorrhagic shock or exsanguination often occurs within the first few hours. The mainstay of treatment therefore involves early identification of patients at risk for hemorrhagic shock in order to provide blood products and control of the bleeding source if necessary. The intended scope of this review is to identify and assess combinations of predictors informing therapeutic decision-making for clinicians during the initial trauma assessment. The primary objective of this systematic review is to identify and critically assess any existing multivariable models predicting significant traumatic hemorrhage that requires intervention, defined as a composite outcome comprising massive transfusion, surgery for hemostasis, or angiography with embolization for the purpose of external validation or updating in other study populations. If no suitable existing multivariable models are identified, the secondary objective is to identify candidate predictors to inform the development of a new prediction rule.

**Methods:**

We will search the EMBASE and MEDLINE databases for all randomized controlled trials and prospective and retrospective cohort studies developing or validating predictors of intervention for traumatic hemorrhage in adult patients 16 years of age or older. Eligible predictors must be available to the clinician during the first hour of trauma resuscitation and may be clinical, lab-based, or imaging-based. Outcomes of interest include the need for surgical intervention, angiographic embolization, or massive transfusion within the first 24 h. Data extraction will be performed independently by two reviewers. Items for extraction will be based on the CHARMS checklist. We will evaluate any existing models for relevance, quality, and the potential for external validation and updating in other populations. Relevance will be described in terms of appropriateness of outcomes and predictors. Quality criteria will include variable selection strategies, adequacy of sample size, handling of missing data, validation techniques, and measures of model performance.

**Discussion:**

This systematic review will describe the availability of multivariable prediction models and summarize evidence regarding predictors that can be used to identify the need for intervention in patients with traumatic hemorrhage.

**Systematic review registration:**

PROSPERO CRD42017054589

**Electronic supplementary material:**

The online version of this article (doi:10.1186/s13643-017-0480-0) contains supplementary material, which is available to authorized users.

## Background

### Traumatic hemorrhage and its consequences

Significant hemorrhage following a traumatic injury progresses quickly within the first few hours and can result in significant morbidity or mortality [1]. It is responsible for the majority of deaths in the operating room and nearly half of all deaths within the first 24 h following an injury [2]. The early recognition and management of bleeding is paramount as unrecognized or uncontrolled hemorrhage is the leading cause of potentially preventable death among this patient population [3]. Following injury, tissue trauma and systemic hypoperfusion is thought to result in trauma-induced coagulopathy—a “global failure” of the coagulation system characterized by anticoagulation and hyperfibrinolysis [4]. Matters are further complicated by the frequent pathophysiological association of hypothermia, acidosis, and coagulopathy following a major traumatic injury, aptly termed the “triad of death” [5]. These interactions result in an exaggerated bleeding state which, when not lethal, can also lead to massive consumption of blood products and risk of significant morbidity.

Ongoing bleeding that is not rapidly identified and corrected results in a state of global hypoperfusion which in turn can lead to multiple organ dysfunction or failure [6]. The mainstay of treatment therefore involves early identification of patients at risk for hemorrhagic shock in order to provide packed red blood cells, platelets, and clotting factors as well as hemostatic intervention such as embolization or surgery [7]. The importance of appropriate patient stratification within the first minutes to hours of resuscitation cannot be overstated [7].

### Current tools used for prediction of hemorrhage

The Advanced Trauma Life Support (ATLS) guidelines provide an algorithmic approach, adapted worldwide, to the initial assessment and resuscitation of the trauma patient [8]. The guidelines classify traumatic hemorrhage into four distinct classes of increasing severity based on clinical examination and alterations in baseline vital signs. Proposed thresholds for resuscitation with crystalloid fluids or blood products are provided. However, these guidelines have come under greater scrutiny and criticism in recent years. A large database validation using the Trauma Audit and Research Network in the UK demonstrated that the ATLS guidelines overestimate the degree of tachycardia and hypotension associated with increasing blood loss [9]. In other words, significant blood loss can occur insidiously before obvious disturbances in vital signs appear. Similarly, another multicenter database validation using the Trauma Registry of the German Society for Trauma Surgery found that less than 10% of patients could be classified accurately according to the ATLS guidelines [8]. The remaining patients demonstrated conflicting clinical parameters that would not permit for classification into any ATLS class of hemorrhage. It is not surprising then that in a 2012 international survey of ATLS course directors and instructors, only 10.9% of respondents stated that they considered the ATLS classification of hemorrhagic shock to be a “good guide for fluid resuscitation and blood product transfusion” [10].

In recent years, several clinical prediction models for hemorrhage have been proposed. These include the Trauma Associated Severe Hemorrhage (TASH) Score [11] or the Assessment of Blood Consumption (ABC) Score [12] for early prediction of patients requiring massive transfusion. However, the need for massive transfusion does not account for all clinically significant outcomes related to hemorrhage and, when used in isolation, is prone to competing risks bias and survivorship bias [13]. Consider that any patient with significant bleeding may be identified quickly and offered a hemostatic intervention such as embolization or surgery long before meeting blood product utilization thresholds for massive transfusion. For this reason, any prediction model for traumatic hemorrhage should seek to evaluate the totality of clinically significant outcomes related to bleeding in order to minimize bias.

### Why it is important to do this review

While some patients arrive in hospital with an obvious need for early intervention, a subset of the trauma population does not manifest the classical clinical or biochemical extremes at presentation that prompt urgent action. It is this group in particular that is at risk of having their degree of hemorrhagic injury underestimated and therefore requires a systematic, evidence-based approach to early diagnosis. There is literature to suggest that elderly patients are significantly more prone to having massive bleeding missed on primary survey due to the absence of vital sign abnormalities and high incidence of non-cavitary, multi-site bleeding [14]. Unfortunately, there exists little in the way of commonly used alternatives to the ATLS classification for guiding clinical decision-making in traumatic hemorrhage. Much of the existing work in this field focusses primarily on prediction models evaluating the need for massive transfusion as noted previously. However, this fails to capture the totality of clinically relevant interventions for hemorrhage, such as the need for hemostatic surgery or angiography with embolization. While a multitude of predictors have been studied with varying levels of success, there remain no widely adopted, evidence-based guidelines for their collective use. Development of such a decision-making framework to allow for early identification of patients needing interventions for hemorrhage would require a meticulous understanding of the existing clinical prediction literature. The recent Prognosis Research Strategy (PROGRESS) series recommendations note that the current methodological standard for modeling research is quite poor and needs to be improved [15]. Most new publications in this field describe only model development with very few considering external validation of previously developed models or evaluating clinical impact. For the procurement of reliable and clinically useful models, it is recommended that they be developed from large, high-quality datasets and validated externally in a separate population. Therefore, the PROGRESS investigators argue that any new modeling endeavors should begin not from scratch but instead with the systematic identification of existing models and consideration of potential for external validation or modernization.

### Objective

The primary objective of this systematic review is to identify and critically assess any existing multivariable models predicting significant traumatic hemorrhage that requires intervention, defined as a composite outcome comprising massive transfusion, surgery for hemostasis, or angiography with embolization. These are prognostic models intended to predict future events and inform therapeutic decision-making. We will evaluate these models for usefulness, relevance, and the potential for external validation and updating in other study populations. The intended scope of this review is to identify and assess a specific combination of predictors informing therapeutic decision-making for clinicians during the initial trauma assessment. If no existing models meeting our pre-specified criteria are identified, we will then secondarily identify potential candidate predictors for future derivation of a new prediction model.

## Methods/design

### Protocol registration

This systematic review protocol was designed using the Preferred Reporting Items for Systematic Review and Meta-Analyses Protocol (PRISMA-P) checklist [16] as well as the Critical Appraisal and Data Extraction for Systematic Reviews of Modeling Studies (CHARMS) checklist [17]. The protocol has been registered with the PROSPERO International Prospective Register of Systematic Reviews (PROSPERO CRD42017054589)

### Population

We will include all studies examining adult patients, aged 16 years or older presenting to hospital with a traumatic injury. Blunt or penetrating mechanisms of injury involving the thorax, abdomen, or pelvis will be acceptable. Studies evaluating only patients with isolated head injury or isolated limb injuries will be excluded.

### Predictors

Multivariable models will be eligible if they include any predictors typically available to the clinician during the first hour of the trauma assessment in the emergency department. This includes any pre-hospital or in-department variables. For the purposes of this review, these will include lab-based predictors such as the complete blood count, blood gases, and coagulation tests. Any concerns regarding time of availability for a laboratory test will be reviewed with a clinical expert in laboratory medicine to determine eligibility. Clinical predictors will include vital signs and point-of-care cardiopulmonary testing such as heart rate variability. Imaging-based predictors will include focussed assessment with sonography in trauma (FAST) ultrasound or computed tomography (CT) scanning. Scoring or injury classification systems that are applied retrospectively (injury severity score, abbreviated injury scale) will be excluded. Reliance on serial measurements following an admission will be excluded. There is no pre-defined categorization of these variables, and they will be extracted as defined by the study authors. The model is intended to be used at the time of initial trauma assessment and resuscitation in the ED.

### Outcomes

The outcome is defined as the need for any life-saving intervention for traumatic hemorrhage, which serves as a surrogate for clinically significant bleeding. This includes the need for hemostatic surgical intervention, angiographic embolization, or massive transfusion within the first 24 h in hospital. Studies describing surgeries without a documented indication or for non-hemorrhagic reasons, such as hollow viscus perforation, will be excluded. Angiography without embolization will similarly be excluded. There exist a variety of definitions of massive transfusion within the literature including: 10 units over the first 6, 12, and 24 h or complete circulating volume over 24 h [18]. We will include all of these definitions. There is no minimum number or percentage of patients receiving intervention (events) needed for inclusion in this review. The events-per-predictor evaluated will be captured as measure of statistical quality [17].

### Study design

We will include all clinical study designs evaluating predictors for life-saving intervention in traumatic hemorrhage, including randomized studies and cohort studies. There will be no date or language restrictions. Attempts will be made to translate any foreign language papers. This systematic review is primarily intended to identify and evaluate existing multivariable prediction models for our composite outcome of interest. We will include any models evaluating any of our individual outcomes of interest whether developmental, internally validated, or externally validated. Should no appropriate multivariable prediction models for our composite outcome be available, we will secondarily seek to identify potential candidate predictors for a future planned prediction rule derivation study.

### Search strategy and data sources

A comprehensive search strategy was developed in tandem with a health information specialist with expertise in systematic reviews and a clinical expert in the field of trauma. Individual search strategies were created for the EMBASE and MEDLINE databases and are available in Additional files 1 and 2, respectively. We used a combination of MESH terms and derived key words in order to define the trauma population and interventions of interest. We utilized an animal studies filter as suggested by the Cochrane Collaboration [19]. The reference lists of included studies or systematic reviews will be manually reviewed to ensure a comprehensive search. The clinicaltrials.gov registry and Central Cochrane Library databases will also be reviewed to identify unpublished or in-progress studies. In addition, we will search the conference abstracts of the past 3 years for the Trauma Association of Canada, the American Association for the Surgery of Trauma, the Eastern Association for the Surgery of Trauma, and the Trauma, Critical Care and Acute Care Surgery annual meetings.

### Study selection process and data extraction

The literature results will be captured and uploaded to Covidence, a web-based reference manager, for facilitation of screening (online version, Albert Health) [20]. The screening process will involve two independent reviewers for title and abstract screening followed by full-text screening with disagreements resolved by a senior reviewer. Abstracts selected by at least one reviewer will be obtained in full for evaluation.

Data extraction will be performed independently by the two reviewers using a pre-defined and piloted data collection form informed by the CHARMS checklist [17]. Abstracted data will include publication characteristics (title, year of publication, author) and patient and institution demographics (study inclusion criteria, trauma center level, civilian or military population). For multivariable models, we will extract model characteristics such as included model type, predictor variables included, sample size, handling of missing data, model development, validation technique, and performance (calibration, discrimination, sensitivity, specificity, positive predictive value, and negative predictive value) as well as model presentation and interpretation as suggested by the CHARMS guidelines [17]. For studies reporting preliminary bivariable testing as well as multivariable models, we will only report on the final derived model.

### Data synthesis, meta-bias, and confidence

Given the anticipated paucity of literature on this topic, this systematic review is intended to be inclusive, exploratory, and descriptive in nature. If any suitable multivariable prognostic models for our composite outcome of interest are identified, the quality of each identified multivariable model will be described based on items identified in the CHARMS checklist [17], including the appropriateness of the outcomes and predictors, techniques used for variable selection, sample size in terms of events per predictor, handling of missing data, model development, validation technique, and measures of model performance. Quality assessment will be presented descriptively in summary table format and will be used to inform the selection of one or more suitable candidate models for external validation in other study populations. As the primary objective of the review is to identify models for external validation, pooling or meta-analysis is not of interest.

Should no multivariable prediction models for our composite outcome be identified, we will secondarily seek to identify potential candidate predictors for a planned prediction rule derivation study. To inform the selection of candidate predictors for this derivation study, we will tabulate the frequency of use of each candidate predictor among the studies identified in our review, as a measure of potential importance of each predictor. We will also extract measures of predictor-outcome associations (i.e., odds ratios) from any bivariable or multivariable tests involving any of the three outcomes comprising our composite. For predictors used in multivariable models meeting adequate quality criteria across multiple studies, strength of association will be described using individual forest plots. Statistical heterogeneity will be examined using *I* ^2^ statistics [21]. Because significant clinical and statistical heterogeneity is expected across studies (e.g., in terms of definitions of predictors and outcomes, categorization of predictors, the types of models used), pooling of odds ratios using meta-analysis is not anticipated. In the event that meta-analysis is deemed appropriate, pooled measures of association will be calculated using random effects meta-analysis. These results will be used solely to inform the pre-specification of important clinical predictors for consideration in future planned clinical derivation studies.

There are no planned assessments of meta-bias or strength of evidence statements.

## Discussion

The assessment of patients for significant traumatic hemorrhage can prove quite challenging and at times overwhelming. The clinician is tasked with processing inputs from a plethora of clinical, lab-based, and diagnostic imaging-based data sources and utilizing them in a manner that allows for rapid identification of patients at risk of significant bleeding. When such a scenario is rapidly and correctly identified, the patient is able to receive a much needed intervention—often in the form of transfusion, surgery, or angiography. In this systematic review, we will rigorously identify, describe, and summarize the existing literature evaluating predictors of intervention in patients with traumatic hemorrhage.

## Additional files

Additional file 1: Appendix 1 EMBASE search strategy. (DOCX 15 kb)

Additional file 2: Appendix 2 MEDLINE search strategy. (DOCX 15 kb)

## Abbreviations

ATLS: Advanced Trauma Life Support
CT: Computed tomography
FAST: Focussed assessment with sonography in trauma
PRISMA-P: Preferred Reporting Items for Systematic Review and Meta-Analyses Protocol
