# Supplementary material for: Early identification of patients requiring massive transfusion, embolization, or hemostatic surgery for traumatic hemorrhage: a systematic review protocol
Source: Syst Rev. 2017 Apr 13;6:80. doi: 10.1186/s13643-017-0480-0 (PMC5390372; doi:10.1186/s13643-017-0480-0)
Supplement: Supplementary file 1 — Appendix 1 EMBASE search strategy. (DOCX 15 kb) [file 13643_2017_480_MOESM1_ESM.docx]

**OVID EMBASE Search Strategy**

1. trauma.mp
2. (trauma* or polytrauma*).tw
3. exp abdominal injury
4. exp penetrating trauma
5. exp blunt trauma
6. exp crush trauma
7. exp multiple trauma
8. exp traumatic amputation
9. exp traumatic shock
10. or/1-9
11. h?morrhag*.tw
12. transfus*.tw
13. intervention.tw
14. surger*.tw
15. angiogra*.tw
16. laparotomy.tw
17. thoracotomy.tw
18. estimated blood loss.tw
19. or/11-18
20. predict*.ti
21. model*.ti
22. utility.ti
23. scor*.ti
24. validation.ti
25. or/20-24
26. 10 and 19 and 25
27. animals/not humans/
28. 26 not 27
