# Supplementary material for: Early identification of patients requiring massive transfusion, embolization, or hemostatic surgery for traumatic hemorrhage: a systematic review protocol
Source: Syst Rev. 2017 Apr 13;6:80. doi: 10.1186/s13643-017-0480-0 (PMC5390372; doi:10.1186/s13643-017-0480-0)
Supplement: Supplementary file 2 — Appendix 2 MEDLINE search strategy. (DOCX 15 kb) [file 13643_2017_480_MOESM2_ESM.docx]

**OVID MEDLINE Search Strategy**

1. “wounds and injuries”.mp
2. trauma.mp
3. (trauma* or polytrauma*).tw
4. exp wounds, nonpenetrating
5. exp wounds, penetrating
6. exp multiple trauma
7. exp shock, traumatic
8. exp amputation, traumatic
9. exp blast injuries
10. exp abdominal injuries
11. exp thoracic injuries
12. exp war-related injuries
13. or/1-12
14. h?morrhag*.tw
15. transfus*.tw
16. intervention.tw
17. surger*.tw
18. angiogra*.tw
19. laparotomy.tw
20. thoracotomy.tw
21. estimated blood loss.tw
22. or/14-21
23. predict*.ti
24. model*.ti
25. utility.ti
26. scor*.ti
27. validation.ti
28. or/23-27
29. 13 and 22 and 28
30. (animals not humans.sh)
31. 29 not 30
